# Complications Associated with the Use of Expandable Gases in Vitrectomy

**DOI:** 10.1155/2018/8606494

**Published:** 2018-11-18

**Authors:** Piotr Kanclerz, Andrzej Grzybowski

**Affiliations:** ^1^Hygeia Clinic, Gdańsk, Poland; ^2^Department of Ophthalmology, University of Warmia and Mazury, Olsztyn, Poland; ^3^Institute for Research in Ophthalmology, Foundation for Ophthalmology Development, Poznan, Poland

## Abstract

Intraocular gases have been used in vitreoretinal surgery for over 40 years. The aim of this study was to review the complications related to the use of expandable gases in vitrectomy and their management. A PubMed, Cochrane Library, and Embase search was conducted using the terms “intraocular gas” and “vitrectomy for retinal detachment.” Of the articles retrieved by this method, all publications in English and abstracts of non-English publications were reviewed. Intraocular pressure elevation was reported in up to 58.9% patients after vitrectomy with expandable gas administration for retinal detachment. Vitreoretinal surgery is known to induce cataract development. With that, cataract progression is associated with lens exposure to intraocular gas, the duration of such exposure, patient's age, and the magnitude of vitreous removal. With intraocular gas, the posterior surface of the lens becomes a strongly refractive factor, resulting in high myopia and temporary vision impairment. Other complications related to the use of expandable gases include anterior chamber and subconjunctival gas displacement. Single reports on subretinal and cranial gas migration were published. In vitrectomy for uncomplicated retinal detachments, attempts to shift from expandable gases towards air are observed. Nevertheless, gas tamponade remains a reasonable choice for patients suffering from retinal detachment.

## 1. Introduction

Intraocular gases are one of the most useful adjuncts in vitreoretinal surgery and have been used as a substitute for air for over 40 years [1]. Due to their lower water solubility than nitrogen, pure sulfur hexafluoride (SF_6_), hexafluoroethane (C_2_F_6_), and perfluoropropane (C_3_F_8_) will expand when injected into the eye. Their surface tensions prevent fluid movement into retinal breaks, supporting physiological removal of fluid from the retinal space and allowing chorioretinal adhesions. A recent study revealed that intraocular gases have been applied regularly in the years of 1998–2013 and use of C_2_F_6_ has increased compared to C_3_F_8_ [2]. With that, pars plana vitrectomy is becoming the procedure of choice for rhegmatogenous retinal detachment (RRD) [2].

The aim of this study was to review the complications related to application of expandible gases in vitrectomy and possible alternatives.

## 2. Methods

PubMed, the Cochrane Library, and Embase were the main resources used to search the medical literature search. An extensive search was performed to identify the use and complications of intraocular gases in retinal detachment surgery as reported up to May 31, 2018. The following keywords were used in various combinations: *retinal detachment*, *vitrectomy*, *intraocular gas*, *sulfur hexafluoride*, *SF*_*6*_, *perfluoromethane*, *CF*_*4*_, *perfluoroethane*, *hexafluoroethane*, *C*_*2*_*F*_*6*_, *perfluoropropane*, *octafluoropropane*, *C*_*3*_*F*_*8*_, and *complications.* The reference lists of identified publications were also considered potential sources of relevant articles. Studies were critically reviewed to create an overview and guidance for further search. Only articles having English-language abstracts were included. No attempts to discover unpublished data were made. In addition to the search, selected chapters from relevant textbooks were included if necessary.

## 3. Results

The search revealed 1980 records, and after excluding duplicates and studies without English abstracts, 1369 records were screened. We identified 60 publications that evaluated the use of intraocular gases for retinal detachment surgery and complications related to their use. The earliest studies date the 1970s, but more than half of the publications were released after 2000. One review relevant to the topic was found in the Cochrane Database of Systematic Reviews [3]. Early research focused on gas pharmacokinetics, while more recent papers tended to evaluate the possibility of replacing expandable gases with air. The largest database study reported the outcomes of RD surgery in 3,403 eyes [4], while patient safety incidents were analyzed in 38,789 vitreoretinal procedures [5].

## 4. Gases Used in Retinal Detachment Surgery

The ideal gas for vitreoretinal surgery should be nontoxic, inert, insoluble in the aqueous humor, and have a lower water solubility than nitrogen [6]. SF_6_ and perfluorinated short-chain carbon compounds are used (C_2_F_6_ and C_3_F_8_), and when injected into the eye, they undergo three phases before resorption: expansion, equilibration, and dissolution [7, 8]. First, the intraocular gas volume rises as the nitrogen diffusion rate into the bubble is greater than the dissolution of the gas into the surrounding tissue fluid compartment. Second, the concentration of nitrogen in the bubble is equilibrated with the bloodstream, and a small amount of expandable gas diffuses out of the eye. Finally, when the partial pressure of all gases in the bubble equals that in the fluid compartment, the dissolution begins [8]. The water solubility decreases as the carbon chain is elongated. For example, a 0.4 cc gas bubble in rabbit models disappears on average in 6, 16, and 28 days for CF_4_, C_2_F_6_, and C_3_F_8_, respectively [9]. The expandable gases' expansive properties may be diminished or eliminated when diluted with air, subsequently increasing the outward diffusion [10]. The gas concentration and its half-life are linearly correlated [11].

Clinically, the longevity of a gas tamponade may differ from a theoretical model. The gas bubble duration is greater in 20-gauge vitrectomy than in microincision vitrectomy surgery [12, 13]. Such results are associated with insufficient tightness in any unsutured 23-gauge sclerotomy, causing early postoperative microleakage. The half-life of intraocular gases is longer in phakic eyes than in pseudophakic/aphakic eyes, presumably due to increased convection in the vitreous cavity of pseudophakic/aphakic eyes, which can accelerate the absorption rate [7, 14, 15]. With that, longer gas duration is correlated with an increased axial length, vitreous presence, lower aqueous turnover, and blood flow [8, 14, 16]. A survey of vitreoretinal surgeons reported that the clinical longevity of a gas bubble after a complete air-gas exchange is 13–24 days for SF_6_, 28–44 days for C_2_F_6_, and 59–79 days for C_3_F_8_ [14].

## 5. Complications Related to the Use of Intraocular Gases

### 5.1. Gas Migration

Anterior chamber (AC) migration of intravitreal gas is a potential complication, which might occur even in phakic eyes with no significant zonular dehiscence or phacodonesis [17, 18]. Intraoperative gas migration into the AC hampers visualization of the posterior segment, and careful insertion of the tamponade agent without overpressurizing the globe is recommended to prevent this complication. To remove the gas from the AC and prevent further gas prolapse, it might be necessary to insert an ophthalmic viscoelastic device (OVD). In some cases, the OVD might be left inside the eye at the conclusion of surgery if migration still occurs; however, such approach necessitates careful IOP control and postoperative administration of hypotensive agents. Long-term presence of gas bubbles in the AC results in corneal edema and bullous keratopathy [19]. The gas is not toxic to the endothelium itself; however, contact between the gas and corneal endothelium results in decreased endothelial cell nutrition [20]. With that, pars plana vitrectomy itself results in a mild decrease in endothelial cell count [21]. Gas in the AC is usually managed by dilating the pupil and placing the patient face down, to allow the bubbles return to the vitreous compartment, so that the endothelium is bathed by aqueous [22].

Subconjunctival gas migration is another potential complication, particularly in microincision vitreous surgery. Gas displacement might occur intraoperatively due to trocar-associated leakage and postoperatively as a result of inadequate sclerotomy closure. In long-term, gas leakage can result in reduction of intraocular gas volume and retinal tamponade, thus influencing the retinal reattachment rate. However, in most of the cases of minor leakage no treatment is required.

Subretinal gas migration is possible particularly in eyes with optic nerve colobomas or large optic pit [23]. Imperfection in tissue overlying cavitary optic disc, faulty interconnections between the vitreous cavity, subarachnoid, and subretinal spaces, and pressure variations of cerebrospinal fluid play a critical role in subretinal gas displacement. Case reports on cranial migration of intraocular gas in the early postoperative period were published [24, 25]. Subsequently, the patients developed nausea, vomiting, and mental status changes. No surgical intervention was deemed necessary, and short-interval clinical follow-up and serial computed tomography scans were recommended. The intracranial gas gradually resolved spontaneously and so did the mental status changes; however, the vision was lost due to altered tamponade properties.

### 5.2. Raised Intraocular Pressure (IOP)

IOP increase in eyes with intraocular tamponade is a common postoperative complication reported in up to 58.9% of eyes [26, 27]. Elevated IOP after vitrectomy may cause optic nerve damage, retinal ischemia, and subsequent visual loss. The mechanism can be open angle, closed angle, or both. In open-angle mechanism, IOP elevation is due to intraocular gas expansion. Closed-angle cases are less common but are usually a result of anterior displacement of the iris-lens diaphragm and iridocorneal apposition with or without pupillary block.

Studies reporting the incidence of IOP elevation after vitrectomy with gas tamponade are presented in Table 1. Expandable gases are supplied with different concentrations ranging from 20% to 100%, in single-use or multiple-use systems and in low-pressure or high-pressure containers with reducers. In practice, the final concentration used during vitrectomy is at the surgeon's discretion [14]. Interestingly, vitreoretinal surgeons commonly admit to having problems with incorrect gas concentration, and postsurgical IOP elevation is associated with high gas concentrations [35]. Other risk factors include advanced patient age and concomitant circumferential scleral buckling [28, 32]. In older patients, increased risk of IOP elevation is related to decreased ocular elasticity and poorer aqueous outflow, while circumferential scleral buckles decrease outflow by elevating the episcleral venous pressure [32, 36]. The incidence of hypertony is also higher in 20-gauge vitrectomy compared to transconjunctival sutureless vitrectomy, as nonsutured sclerotomies allow a free passage of air/gas if IOP is elevated [37, 38].

Chen noted that, in the majority of cases, IOP elevation is transient with the highest mean values 2–4 hours postoperatively and lasts for up to 1 week after surgery [33]. Elevated IOP potentially can be more dangerous in eyes with preexisting optic nerve damage, i.e., in glaucoma or atherosclerosis-related ischemia. Mittra et al. suggested prophylactic use of antihypertensive topical medication in patients with long-lasting gas tamponade, as in their studies they were necessary to prevent IOP elevation [34, 39]. It might be questioned whether prophylactic treatment should be recommended in all cases. We recommend vitreoretinal surgeons to benchmark their complication rate and adjust their surgical techniques and postoperative regimen.

When faced with high IOP due to an enlarging gas bubble, topical antihypertensive and systemic medications are recommended. Intravenous mannitol is known to work suboptimally in vitrectomized eyes, and several agents might be required to lower IOP. In some cases, it might be necessary to tap the gas in the outpatient clinic or operating room [40].

### 5.3. Cataract Development and Patient Positioning

Cataract is a common complication of vitreoretinal surgery, which develops due to inhibited diffusion of nutrients impeding proper lens metabolism [10]. Exposure to intraocular gases additionally increases retrolental oxygen levels, resulting in development of lens opacities. The severity of cataract progression correlates with the longevity of intraocular tamponade [41]. Cataracts that develop following expandable gas administration manifest as feathery formations, posterior capsule opacities, or vacuoles that are more intense in the superior part of the lens. Singh and associates noted that 18.8% of patients with intraocular gas tamponade develop cataract within three months after surgery [12]. Jackson et al. found that 51.8% of phakic patients after vitrectomy with different gas tamponade agents have subsequent phacoemulsification cataract surgery in the following 6 months [4]. Importantly, extended vitrectomy with surgical posterior vitreous detachment and anterior vitreous removal additionally increases the risk of cataract development [42]. Thompson et al. reported a more pronounced advancement in nuclear sclerosis in older age groups [43]. Postoperatively, the first symptom of nuclear sclerosis might be a myopic shift in refraction [44].

In order to decrease the contact between the gas bubble and the lens, patients might be instructed to avoid supine positioning [10]. Prone positioning might be indicated to support hole closure, as most retinal breaks develop between the posterior vitreous base and the equator [45]. With that, face-down positioning prevents forward movement of the lens-iris diaphragm caused by anterior pressure of the gas bubble, particularly in cases of floppy or atrophic iris [46–49]. Interestingly, in a study by Otsuka et al., prone position was maintained only on the day of surgery, followed by supine positioning for 7 days, and resulted in similar outcomes compared to strict prone positioning [45]. Nevertheless, it might be concluded that in general, patients with intraocular expandible gas should avoid supine positioning. Cataract development is associated with the duration of lens exposure to gas, patient's age, and the magnitude of vitreous removal.

### 5.4. Other Complications

The presence of intraocular gases results in light scattering on the interface between the gas bubble and adjacent intraocular fluid [10, 42]. Due to the high difference in refractive indexes between the gas and the lens, the posterior surface of the lens becomes a strongly refractive factor. Intraocular gases induce high myopia up to 50 D, which progressively diminishes as the size of the bubble decreases [50]. Patients should be informed about the reasons for vision impairment after intraocular gas administration, and it should be taken into consideration when establishing the treatment plan in monocular individuals. Variations in atmospheric pressure affect the total volume of the gas bubble. Thus, safety of patients with intraocular gas is endangered by air travel, particularly in eyes without a scleral buckle [51, 52].

Another potential complication of vitreoretinal surgery is hypotony. Although the incidence of hypotony is significantly lower in eyes with air/gas tamponade than in cases with no tamponade [53], it was reported after 31% of vitrectomies with C_3_F_8_ applied [54]. In most cases hypotony recovers without persistent damage; however, it does impose potential risks, i.e., increased reoperation rate [53].

In experimental studies, Doi and associates noted thinning or disappearance of the outer plexiform layer of rabbits' superior retina after intravitreal administration of 0.4 ml·C_3_F_8_ [55]. This finding was presumably associated with mechanical damage of the retina related to the contact with the expansive gases, rather than the toxicity of the vitreous substitute. In vivo studies did not confirm the influence of gas tamponade on retinal layer segmentation; however, reduction in the ganglion cell and outer retinal layers was found in eyes with long-term silicone oil tamponade [56].

## 6. Alternatives to Expandable Gases in Retinal Detachment Surgery

In addition to possible complications associated with long-acting gases, other disadvantages include their cost, the time, and workload required to acquire and store them and additional surgical time for dilution and administration. Several studies assessed the utility of air tamponade for eyes undergoing vitreoretinal surgery [57, 58]. A significant advantage of applying air compared to expandible gases is a shorter prone-positioning and recovery period. Tan and associates presented that patients with complete air tamponade had a similar vitrectomy success rate compared to 20% SF_6_, but only in RRDs with upper quadrants involved [59]. Zhang et al. reported similar success rate RRDs with superior retinal breaks in eyes with partial and complete air tamponade [60]. In other studies, air was used exclusively for tamponade in vitrectomy for RD, resulting in the similar reattachment rate in inferior breaks as well [57, 58]. Nevertheless, the majority of primary uncomplicated RRDs remain treated with expandible gas tamponade.

As air does not provide a long-term tamponade, it cannot be considered in eyes with recurrent RDs, in primary RDs that are associated with a varying intensity of proliferative vitreoretinopathy, or growth of fibrous tissue [3]. Schwartz et al. in the Cochrane Database for Systematic Reviews revealed that only the use of perfluoropropane (C_3_F_8_) or silicone oil is a reasonable choice for most patients with RD associated with proliferative vitreoretinopathy [3]. With that, long-lasting gas tamponade with C_2_F_6_ or C_3_F_8_ might be recommended for RRDs with inferior breaks or with giant retinal tears [61, 62].

## 7. Conclusions

The use of intraocular gases can result in postoperative intraocular pressure elevation, cataract formation, gas migration, and temporary vision impairment due to the a high difference in refractive indexes between the gas and the lens. In vitrectomy for uncomplicated retinal detachments, attempts to shift from expandable gases towards air are observed. Nevertheless, gas tamponade remains a reasonable choice for patients suffering from retinal detachment.

## Figures and Tables

**Table 1:** The incidence of raised intraocular pressure after vitrectomy with gas tamponade.

Author	Incidence of IOP elevation (%)	Definition of IOP elevation	Type of surgical intervention	Gas	Risk factors for IOP elevation
Abrams et al. [28]	45	≥30 mmHg in the early postoperative period	PPV with SF_6_ to reform soft eyes	SF_6_ 20–100%	Eyes with postoperative fibrinous anterior chamber exudates, 100% gas concentration
Chang et al. [26]	58.9	>22 mmHg within the first postoperative week	PPV for complicated retinal detachment	C_2_F_6_–C_3_F_8_ in various concentrations	N/A
The Silicone Study Group [29]	8.7	≥30 mmHg at any postoperative visit	PPV in eyes with proliferative vitreoretinopathy and prior vitrectomy	C_3_F_8_ 14%	N/A
The Silicone Study Group [30]	6.1	≥30 mmHg at any postoperative visit	PPV in eyes with proliferative vitreoretinopathy and prior vitrectomy	SF_6_ 20%	N/A
Wong et al. [31]	21.7	>30 mmHg on postoperative Day-1	PPV with or without phacoemulsification cataract surgery	C_3_F_8_ 16%	N/A
Wong et al. [31]	20.4	>30 mmHg on postoperative Day-1	PPV with or without phacoemulsification cataract surgery	SF_6_ 30%	N/A
Chen and Thompson [32]	43	>25 mmHg in early postoperative period	PPV with or without scleral buckling	SF_6_ 10–30% or C_3_F_8_ 5%–35%	Increasing patient age; expansile gas concentrations; use of C_3_F_8_; circumferential scleral buckles
Chen [33]	52	>30 mmHg within 1 week after surgery	PPV for macular hole surgery	C_3_F_8_ 14%	N/A
Mittra et al. [34]	52.4 (>25 mmHg) 28.6 (>30 mmHg)	Elevation 4–6 hours postoperatively	PPV	SF_6_ 18%–20% or C_3_F_8_ 12%–16%	N/A
Wong et al. [5]	0.5–1.3	N/A	Vitreoretinal surgery	N/A	N/A

Abbreviations: IOP, intraocular pressure; PPV, pars plana vitrectomy. The study by Wong et al. [5] presenting the incidence of IOP elevation in all vitreoretinal procedures is presented for comparative purposes.

## Appendix

### A. Methods for Literature Search

Literature searches of the PubMed, Embase, and Cochrane databases were conducted in May 2018; the search strategies are as follows. Specific limited update searches were conducted after May 2018.

#### A.1. PubMed Searches (Publication Date 1/10/11–5/31/2018)

((“retinal detachment”[MeSH]) OR (“vitrectomy”[MeSH])) AND ((“gas”[MeSH]) OR (“sulfur hexafluoride”[MeSH]) OR (“perfluoromethane”[MeSH]) OR (“perfluoroethane”[MeSH]) OR (“perfluoropropane”[MeSH]) OR (“octafluoropropane”[MeSH]) OR (“SF6”[Title/Abstract]) OR (“CF4”[Title/Abstract]) OR (“C2F6”[Title/Abstract]) OR (“C3F8”[Title/Abstract])). 803 references.

#### A.2. Cochrane Searches (Publication Date 1/10/11–5/31/2018)

MeSH descriptor Retinal Detachment explode all trees in Cochrane Database of Systematic Reviews. 23 references.

MeSH descriptor Vitrectomy explode all trees in Cochrane Database of Systematic Reviews. 15 references.

#### A.3. Embase Searches (Publication Date 1/10/11–5/31/2018)

((^*∗*^“retinal detachment”^*∗*^/de OR ^*∗*^“retinal detachment”^*∗*^) OR (^*∗*^“vitrectomy”^*∗*^/de OR ^*∗*^“vitrectomy”^*∗*^)) AND ((^*∗*^“gas”^*∗*^/de OR ^*∗*^“gas”^*∗*^) OR (^*∗*^“sulfur hexafluoride”^*∗*^/de OR ^*∗*^“sulfur hexafluoride”^*∗*^) OR (^*∗*^“perfluoromethane”^*∗*^/de OR ^*∗*^“perfluoromethane”^*∗*^) OR (^*∗*^“perfluoroethane”^*∗*^/de OR ^*∗*^“perfluoroethane”^*∗*^) OR OR (^*∗*^“perfluoropropane”^*∗*^/de OR ^*∗*^“perfluoropropane”^*∗*^) OR (^*∗*^“octafluoropropane”^*∗*^/de OR ^*∗*^“octafluoropropane”^*∗*^) OR (^*∗*^“SF6”^*∗*^/de OR ^*∗*^“SF6”^*∗*^) OR (^*∗*^“CF4”^*∗*^/de OR ^*∗*^“CF4”^*∗*^)OR (^*∗*^“C2F6”^*∗*^/de OR ^*∗*^“C2F6”^*∗*^) OR (^*∗*^“C3F8”^*∗*^/de OR ^*∗*^“C3F8”^*∗*^)) 1139 references.
